# Joint elasticity produces energy efficiency in underwater locomotion: Verification with deep reinforcement learning

**DOI:** 10.3389/frobt.2022.957931

**Published:** 2022-09-08

**Authors:** Chu Zheng, Guanda Li, Mitsuhiro Hayashibe

**Affiliations:** Neuro-Robotics Laboratory, Department of Robotics, Graduate School of Engineering, Tohoku University, Sendai, Japan

**Keywords:** underwater, snake robot, joint elasticity, energy efficiency, deep reinforcement learning, curriculum learning

## Abstract

Underwater snake robots have received attention because of their unique mechanics and locomotion patterns. Given their highly redundant degrees of freedom, designing an energy-efficient gait has been a main challenge for the long-term autonomy of underwater snake robots. We propose a gait design method for an underwater snake robot based on deep reinforcement learning and curriculum learning. For comparison, we consider the gait generated by a conventional parametric gait equation controller as the baseline. Furthermore, inspired by the joints of living organisms, we consider elasticity (stiffness) in the joints of the snake robot to verify whether it contributes to the generation of energy efficiency in the underwater gait. We first demonstrate that the deep reinforcement learning controller can produce a more energy-efficient gait than the gait equation controller in underwater locomotion, by finding the control patterns which maximize the effect of energy efficiency through the exploitation of joint elasticity. In addition, appropriate joint elasticity can increase the maximum velocity achievable by a snake robot. Finally, simulation results in different liquid environments confirm that the deep reinforcement learning controller is superior to the gait equation controller, and it can find adaptive energy-efficient motion even when the liquid environment is changed. The video can be viewed at https://youtu.be/wpwQihhntEY.

## 1 Introduction

With the development of robotics technology, numerous underwater mobile robots have been recently conceived and prototyped for underwater oil and gas exploration, ocean observation, rescue work, and ocean science research (Chutia et al. (2017); Khatib et al. (2016)). Without carrying human drivers, these robots can greatly reduce the risk of accident and the cost, thereby improving their performance with longer time of exploration in the water.

The use of a snake robot as a typical mobile robot has attracted the attention of researchers, owing to its unique undulatory locomotion. In addition, the flexible body of snake robots allows them to easily move underwater. In Kelasidi et al. (2015), a simulation study was performed to compare the total energy consumption and cost of transportation between underwater snake robots and remotely operated vehicles. The simulation results showed that, regarding the cost of transportation and total energy consumption, the underwater snake robots are more energy-efficient for all the evaluated motion modes compared with the remotely operated vehicles.

The first snake robot was introduced by Hirose in the 1970s (Hirose (1993). Since then, researchers have developed a large variety of snake robots. A review of land-based snake robots shows that most studies have been focused on locomotion over flat surfaces, but a growing trend has emerged toward locomotion in more challenging environments (Liljebäck et al. (2012)). Unlike land-based snake robots, only a few swimming snake robots have been developed, including the eel robot REEL (Melsaac and Ostrowski (1999); McIsaac and Ostrowski (2002)), lamprey robot AmphiBot (Crespi et al. (2005); Crespi and Ijspeert (2006); Porez et al. (2014)), amphibious snake robot ACM 5 (Ye et al. (2004); Yamada (2005)), and an underwater snake robot with thrusters (Kelasidi et al. (2016)).

Despite existing developments, it remains challenging to generate robust and efficient gaits for snake robots, owing to their special mechanical structure and redundant degrees of freedom. The model-based method is a common control architecture method for snake robots based on either kinematic or dynamic models for control (Ostrowski and Burdick (1996)). Although the model-based method can quickly generate the best gait in simulations for a given robot, its approximate analytical solution presents limitations. First, the control performance deteriorates when the model becomes less accurate. As the control of a snake robot depends on the operation frequency, small frequency fluctuations can lead to large changes in the gait, especially in real underwater environments. Second, model-based controllers are not always suited for interactive modulation under unknown and varying environments Crespi and Ijspeert (2008).

Previous studies on swimming snake robots have been focused on two motion patterns: lateral undulation and eel-like motion Kelasidi et al. (2015). The gait equation that describes the joint angles over time is generally used to define the motion patterns of the snake robots. Complex and different motion patterns can be obtained by setting just a few parameters. However, this parameterized gait may be limited by speed to find the best combination of parameters for a given situation. One possible approach is to find the best parameters by searching a grid of gait parameters with fixed intervals through simulations. However, the difference between simulated and real environments can lead to biases, being difficult for the grid search method to provide the best combination of parameters in practice. In Kelasidi et al. (2015), empirical rules were used to choose the gait parameters considering both the desired forward velocity and power consumption of the robot. However, optimizing gait parametrically is limited to the selection of a few specific parameters, limiting the optimization scope.

Diverse problems in robotics can be naturally formulated as reinforcement learning problems. Reinforcement learning offers a framework and a set of tools for designing sophisticated, hard-to-engineer behaviors (Kober et al. (2013)). In particular, deep reinforcement learning (DRL) has been studied to control snake robots. In Bing et al. (2020b), target-tracking tasks for a snake robot were solved using a reinforcement learning algorithm. In Bing et al. (2020a), DRL was applied to improve the energy consumption of snake robot motion using a slithering gait for the ground locomotion. In terms of natural animal-like motor learning, synergetic motion in redundancy could be generated through DRL along with the increase in the energy efficiency during legged locomotion (Chai and Hayashibe, 2020). In this study, we apply DRL to investigate the impact of joint elasticity on improving the energy efficiency of underwater snake locomotion. In designing the reward function, we introduce the concept underlying curriculum learning (Bengio et al. (2009): humans and animals learn much better when the examples are not randomly presented but organized in a meaningful order and gradually presenting with more concepts with increasing complexity. At the beginning, the reward function is relatively simple. After a certain number of training epochs, the reward function changes to a more complex target task.

First, we demonstrate that DRL can be used for a snake robot to move underwater with more efficiency than when using a conventional gait equation controller based on grid search to determine the optimal parameters. The DRL controller is trained by proximal policy optimization, which is a typical model-free DRL approach. We also evaluate the relation between the average velocity and output power of different types of gaits generated by the DRL and gait equation controllers. The result shows that the DRL controller achieves the highest performance for an underwater locomotion.

Second, we change the joint elastic attributes of a snake robot considering the structure of different organisms. It is verified if the locomotion in different environments requires different joint stiffnesses for energy efficiency, e.g., in walking Farley et al. (1998); Xiong et al. (2015). For a human jump motion, joint elasticity induced by the collaboration of muscles and tendons enables high-power movement as the elastic element can store the potential energy (Otani et al., 2018). Similarly, we consider the elasticity in the joints of a snake robot to resemble the joints of real animals. We evaluate an underwater locomotion with different joint elasticities considering the average velocity and the energy efficiency. The results show that an appropriate joint elasticity allows moving with higher energy efficiency, but for the control solution to take advantage of it, it should be well explored.

Finally, we report experiments in different liquid environments. In addition to water, snake robots can be deployed to extreme environments such as a marine oil-spill scenario. The simulation results demonstrate that the DRL controller retains its superiority even when the fluid environment changes and that an appropriate joint elasticity can still improve the energy efficiency of swimming.

The remainder of this article is organized as follows. The simulation models of the snake robots are introduced in Section 2. The gait equation controller and DRL controller used to generate the gait for snake robots are described in Section 3. Section 4 compares and analyzes the energy efficiency of snake robots with different gaits. Section 5 presents the conclusions of this study.

## 2 Simulation method and energy efficiency

### 2.1 Simulation method

**FIGURE 1:**
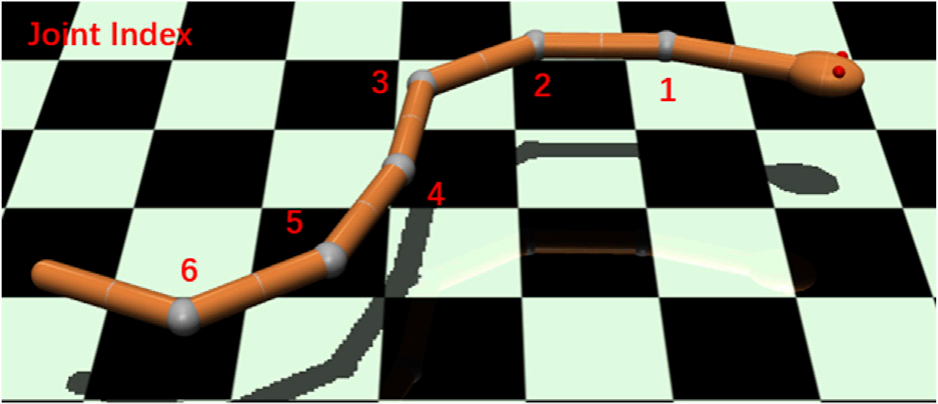
Snake robot in the simulation environment (MuJoCo). Red numbers show the joint index.

We used the MuJoCo physics simulation engine to model and simulate our underwater snake robot. MuJoCo provides fast and accurate dynamic simulations for robotics, biomechanics, and medicine (Todorov et al. (2012)). In the previous study by Li et al., (2021), we proposed a simulation framework for soft-bodied robot underwater locomotion in MuJoCo. In this work, we focus on the rigid-bodied underwater snake robot with different joint elasticity levels to reveal the impact on energy efficiency. The main simulation environment is in water with a density of 1,000 kg/m^3^ and dynamic viscosity of 0.0009 Pa⋅ s. We also carried out experiments in different liquid environments, with reference to propylene (density of 514 kg/m^3^ and dynamic viscosity of 0.0001 Pa⋅ s) and ethylene glycol (density of 1097 kg/m^3^ and dynamic viscosity of 0.016 Pa⋅ s).As shown in Figure 1, the snake robot in the simulation environment has seven links connected by six rotational joints with one degree of freedom per joint. Each link has a length of 0.1 m and diameter of 0.02 m, and the total mass of the robot is 0.25 kg. To eliminate the effects of buoyancy, a uniform density of 1,000 kg/m^3^, which is the same as the liquid environment, is set for all components of the model.

The snake robot can move forward underwater by controlling each joint, which can rotate in the range of [−90°, 90°]. The force of each motor is limited within the range [-1 N, 1 N], and its gear ratio is 0.1. Therefore, the actuator torque range is [−0.1 N⋅ m, 0.1 N⋅ m] obtained by multiplying the actuator force by the gear ratio. For the gait equation controller, the servo motor is used because it outputs the joint angle directly. On the other hand, the DRL controller makes exploration directly for the motor torque space. The physical parameters of the joint may affect the gait performance.

In order to explore the effect of joint stiffness on the energy efficiency of the snake robot, we verify the effect of the joint elasticity by varying the stiffness parameter with different settings. For both the gait equation controller and DRL controller, the simulation frequency was 100 Hz, and the control frequency was 25 Hz.

### 2.2 Power efficiency

For a snake robot with *N* joints, instantaneous power consumption *P* is calculated as

$$P=\overset{N}{\underset{j=1}{\sum }}|\tau_{j}{\dot{\phi }}_{j}|,$$ (1)

where *τ*_*j*_ is the torque of the actuator *j*, and $\dot{\phi_{j}}$ is the angular velocity of the joint *j*. Average power consumption $\bar{P}$ during a run with *k* steps is calculated as

$$\bar{P}=\frac{1}{k}\overset{k}{\underset{1}{\sum }}\overset{N}{\underset{j=1}{\sum }}|\tau_{j}{\dot{\phi }}_{j}|.$$ (2)

Since

$$\tau_{j}=f_{j}h_{j},$$ (3)

where *f*_*j*_ is the applied force, *h*_*j*_ is the gear constant (i.e., gear ratio of actuator) of the joint *j*, and force *f*_*j*_ applied by the actuator is limited to a maximum of *f*_max_. Normalized power consumption $\hat{P}$ at each time step is calculated as

$$\hat{P}=\frac{1}{N}\overset{N}{\underset{j=1}{\sum }}\frac{\left|f_{j}h_{j}{\dot{\phi }}_{j}\right|}{f_{\max}h_{j}{\dot{\phi }}_{\max}}=\frac{1}{N}\overset{N}{\underset{j=1}{\sum }}\frac{\left|f_{j}{\dot{\phi }}_{j}\right|}{f_{\max}{\dot{\phi }}_{\max}}.$$ (4)

We use $\hat{P}$ for defining the reward in DRL.

## 3 Controller design

In this section, we introduce the gait equation and DRL controllers. The gait equation controller is a model-based method with a fixed equation and various adjustable parameters. The DRL controller is a model-free method that enables a robot to autonomously discover the optimal behavior through trial-and-error interactions with its environment (Kober et al., 2013). A DRL controller can overcome the limitations to the conventional gait equation and allows exploring various types of gaits.

### 3.1 Gait equation controller

The gait equation controller is formulated as

$$\phi \left(i,t\right)=g\left(i,y\right)A\sin\left(\omega t+\lambda i\right)+\gamma ,$$ (5)

where

$$g\left(i,y\right)=\frac{i}{N}\left(1-y\right)+y.$$ (6)

*ϕ*(*i*, *t*) represents the joint angle at time *t*, with *i* being the joint index and *N* being the number of joints, *g*(*i*, *y*) is a scaling function for the amplitude of joint *i* that allows function (5) to describe a general class of sinusoidal functions and their corresponding snake motion patterns (Kelasidi et al., 2014). Setting *y* = 1 provides lateral undulation, in which the amplitudes of each point of the snake robot are of the same magnitude. Setting *y* = 0 provides an eel-like motion, in which the amplitudes of each point of the snake robot increase from the head to tail.

Moreover, *A* is the serpentine amplitude, *ω* is the temporal frequencies of the movement, *λ* determines the phase shift between the joints, and *γ* is a parameter that controls the steering of the snake robot.

By adjusting *y*, *A*, *λ*, and *ω*, the gait of the snake robot can be changed, and the optimal gait parameters can be found by using a method called grid search. The parameters and ranges for grid search are listed in Table 1, resulting in 32,400 parameter sets. We tested each motion parameter set by running 1,000 steps in the simulations. For each run, we ignored the first 200 steps that were considered as the warm-up time for the snake robot to accelerate and stabilize its swimming gait. The remaining 800 steps were used for calculating the average velocity and energy efficiency.

**TABLE 1:** Parameters used for the grid search.

Parameter	Description	Values	Step length
*A*	Amplitude	[10°, 180°]	10°
*Ω*	Temporal frequency	[0.05, 1]	0.05
*Λ*	Phase	[10°, 180°]	10°
*Y*	Linear reduction	[0.2, 1]	0.2

### 3.2 DRL controller

**FIGURE 2:**
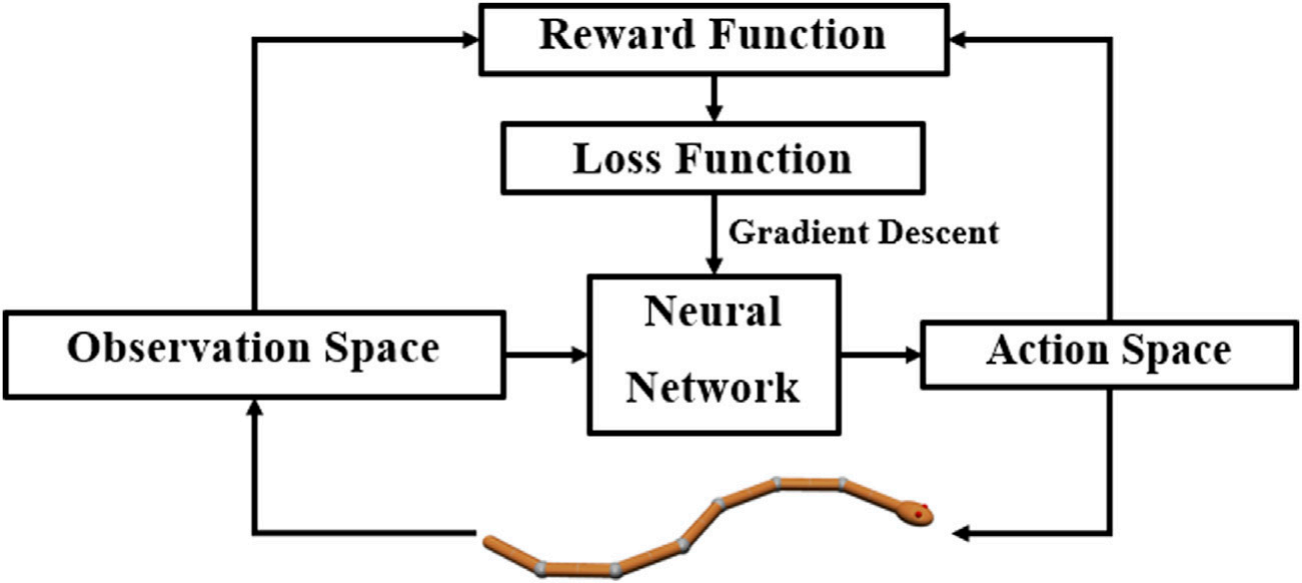
Perception–action–learning loop of the DRL controller. At time *t*, the agent receives the state *s*_*t*_ from the environment. The agent uses its policy to choose an action *a*_*t*_. Once the action is executed, the environment transitions a step, providing the next state, *s*_*t*+1_, as well as feedback in the form of a reward, *r*_*t*+1_. The agent uses knowledge of state transitions, of the form (*s*_*t*_, *a*_*t*_, *s*_*t*+1_, *r*_*t*+1_), to learn and improve its policy.

DRL combines reinforcement learning and deep learning. Reinforcement learning allows robots to learn from their interactions with the environment and autonomously discover and explore the best behavior for a given goal. Deep learning expands reinforcement learning to decision-making problems that were previously intractable, that is, to settings with high-dimensional states and action spaces Arulkumaran et al. (2017). Figure 2 shows the perception–action-–learning loop of the DRL controller.

#### 3.2.1 Algorithm

The leading common policy gradient algorithms are Soft Actor-Critic (SAC) (Haarnoja et al. (2018)), Trust Region Policy Optimization (TRPO) (Schulman et al. (2015)), and Proximal Policy Optimization (PPO) (Schulman et al. (2017)). TRPO is relatively complicated and is not compatible with architectures that include noise or parameter sharing. The PPO algorithm uses a penal to ameliorate the excessively large optimization to obtain better sampling complexity at the basis of the TRPO methods. PPO is an on-policy algorithm, i.e., PPO faces serious sample inefficiency and requires a huge amount of sampling to learn, which is unacceptable for real robot training. But for simulations, PPO shows its superiority compared to SAC. In Xu et al. (2021), the authors defined a 3D environment in Unity to train cart racing agents. They tested the PPO and SAC algorithms in different environments. The authors have experimentally verified that the PPO algorithm has a better performance in the convergence rate and practical results (the average speed of agent) than SAC. So in this work, we trained the neural network using PPO-Clip.

PPO-Clip is one of the primary variants of PPO. PPO-Clip relies on specialized clipping in the objective function to remove incentives for the new policy to get far from the old policy. Algorithm 1 shows the pseudo-code of PPO-Clip.

Algorithm 1 PPO-Clip.

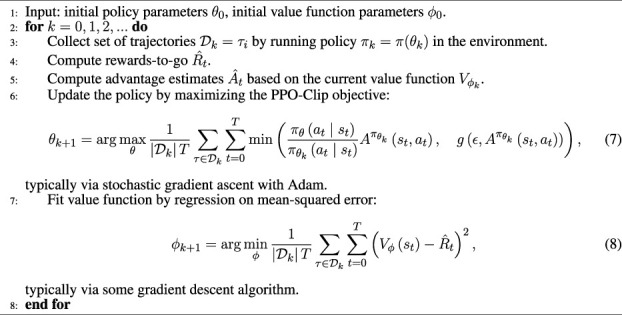

#### 3.2.2 Reward function

In Bing et al. (2020a), an effective and reliable reward function was proposed to simultaneously control the velocity of a snake robot and optimize its energy efficiency. First, a normalized reward allows the robot to maintain its target velocity. The objective is to reach and maintain target velocity *v*_*t*_ by comparing it with the average velocity $\bar{v}$ of the center of mass of the snake robot. The velocity reward is given by

$$r_{v}={\left(1-\frac{\left|v_{t}-\bar{v}\right|}{a_{1}}\right)}^{a_{2}}.$$ (9)

Parameter *a*_1_ = 0.2 influences the spread of the reward curve by defining the *x*-axis intersections with *x* = *v*_*t*_ ± *a*_1_, while *a*_2_ = 5 affects the curve gradient. If $|v_{t}-\bar{v}|=0$ , velocity reward *r*_*v*_ has a maximum value of 1.

Second, the normalized value of the average power efficiency $\hat{P}$ in function (4) is used to determine the power efficiency reward *r*_*P*_ as follows:

$$r_{P}=|1-\hat{P}{|}^{b}.$$ (10)

The parameter *b* = 3 affects the curve gradient.

Finally, the rewards from the velocity (*r*_*v*_) and power efficiency (*r*_*P*_) are combined into the overall reward *r*_1_:

$$r_{1}={\left(1-\frac{\left|v_{t}-\bar{v}\right|}{a_{1}}\right)}^{a_{2}}|1-\hat{P}{|}^{b}.$$ (11)

#### 3.2.3 Observation space

The DRL controller obtains information about the robot and environment from the observational space at each step. Only with suitable and sufficient information, the DRL controller can develop adequate control strategies. The observation space O used to train the snake robots is given by

$$\begin{array}{c} O=\left[A_{h},A_{1},A_{2},A_{3},A_{4},A_{5},A_{6},\right.\\ \left.\theta_{h},\theta_{1},\theta_{2},\theta_{3},\theta_{4},\theta_{5},\theta_{6},\right.\\ \left.Vel_{h}x,Vel_{h}y\right], \end{array}$$ (12)

where *A*_*h*_ is the angular velocity of the head, *A*_1_–*A*_6_ represent the angular velocity of the corresponding joints, *θ*_*h*_ is the rotation angle of the robot head, *θ*_1_–*θ*_6_ represent the rotation angle of the corresponding joints, and *Vel*_*h*_ *x* and *Vel*_*h*_ *y* are the velocities of the robot head on the *x* and *y* axes, respectively.

#### 3.2.4 Action space

Action space A has the same dimensions (six in this study) as the number of actuators in the snake robot because each element in the action space corresponds to each actuator’s torque.

#### 3.2.5 Training configuration

We deployed model training in OpenAI Spinning Up, a DRL framework that can allocate computing resources conveniently. We used a two-layer fully connected network with 256 ReLU (rectified linear unit) functions per layer as the hidden layer of the policy network. The input layer of the policy network has the same dimension as the observation space O , and the output layer has the same dimension as the action space A .

Owing to the complexity of reward function *r*_1_, it is difficult to find the optimal solution directly, as demonstrated as follows. Therefore, we adopted a curriculum learning strategy. Specifically, in the first 2000 epochs, the reward function *r*_2_ was set as

$$r_{2}=cv_{h}-\hat{P},$$ (13)

where *v*_*h*_ is the velocity of the forward motion of the robot head. The parameter *c* = 200 influences the weights of *v*_*h*_ and $\hat{P}$ . The velocity considered in this study is that of the robot head and not that of the center of mass. After 2,000 epochs, the robot moves forward steadily with high speed, and we change the reward function to *r*_1_, and the target velocity decreases by 0.02 every 1,000 epochs, starting from the velocity obtained at epoch 2,000 and decreases until 0.02 m/s. We decrease the target velocity over time instead of increasing it because at the end of 2,000 epochs, the action policy output by the neural network is moving forward at a maximum velocity. If the target velocity in the reward function after 2,000 epochs is small, it is actually a large change for the training target. For curriculum learning, it is important that the task difficulty is gradual, i.e., that the gap between two consecutive tasks is as small as possible.

Main parameters of PPO: the discount factor *γ* is 0.99, the clip ratio is 0.2, the learning rate for the policy optimizer is 0.003, the learning rate for the value function optimizer is 0.001, the GAE parameter *λ* is 0.97, and the KL target is 0.01.

## 4 Results and analysis

**FIGURE 3:**
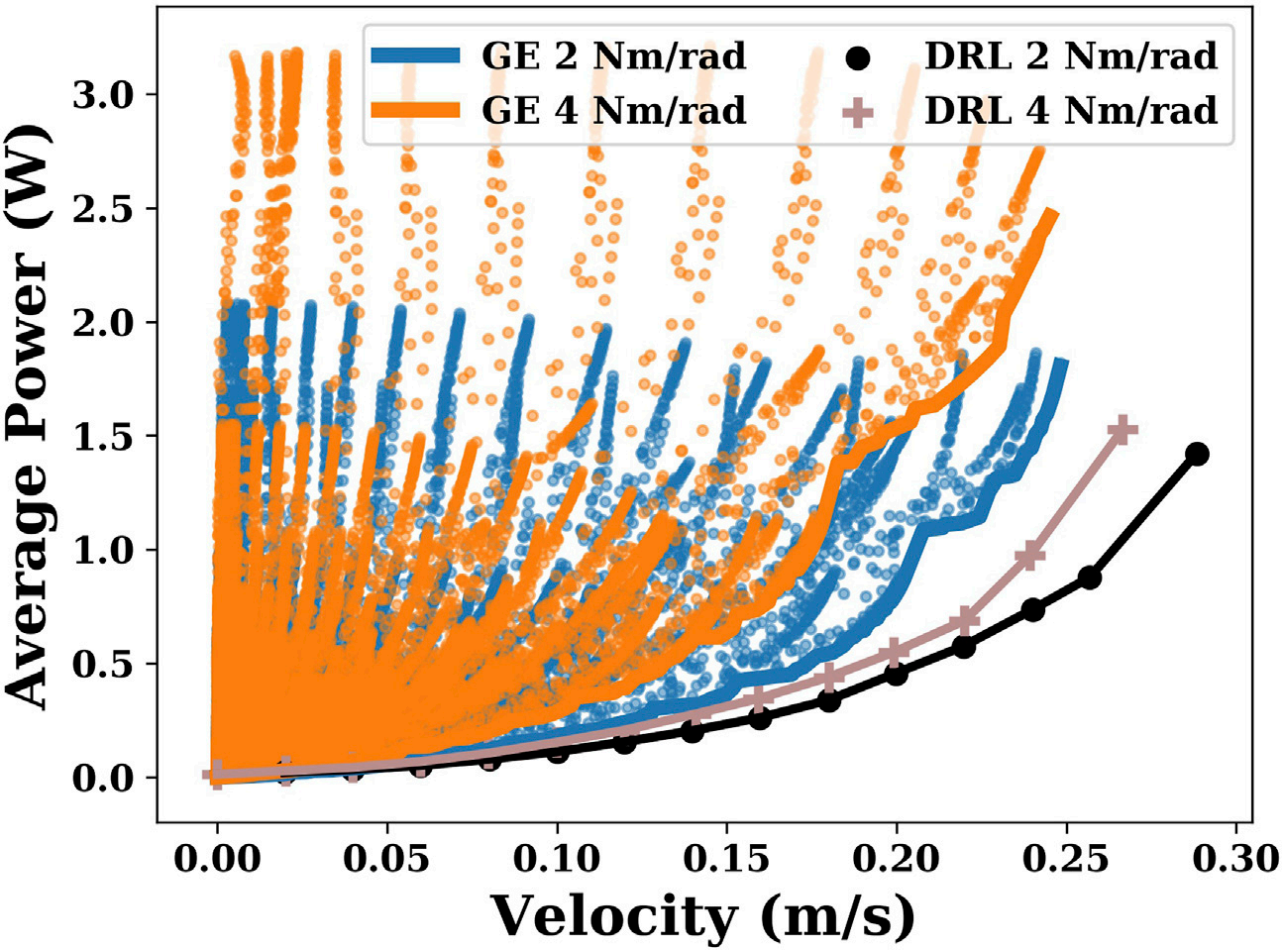
This plot shows the results generated by the gait equation controller and DRL controller when the joint stiffnesses are 2 Nm/rad and 4 Nm/rad. The coordinate of each point represents the velocity and its corresponding average power consumption.

In this section, we compare the differences in energy efficiency between the gait equation controller and DRL controller. Using the gait equation controller, we obtained 32,400 points with different velocities and energy efficiencies in each joint stiffness condition. Using the DRL controller, we obtained several points of different velocities in the range of 0.02 m/s to maximum velocity with a step interval of 0.02 m/s. As shown in Figure 3, when the joint stiffness is 2 Nm/rad and 4 Nm/rad, the DRL controller produces a more efficient gait than the gait equation controller, especially at higher velocities.

### 4.1 Result of the gait equation controller

Utilizing the grid search, the gait equation controller generates 32,400 different gaits in each joint stiffness condition.

**FIGURE 4:**
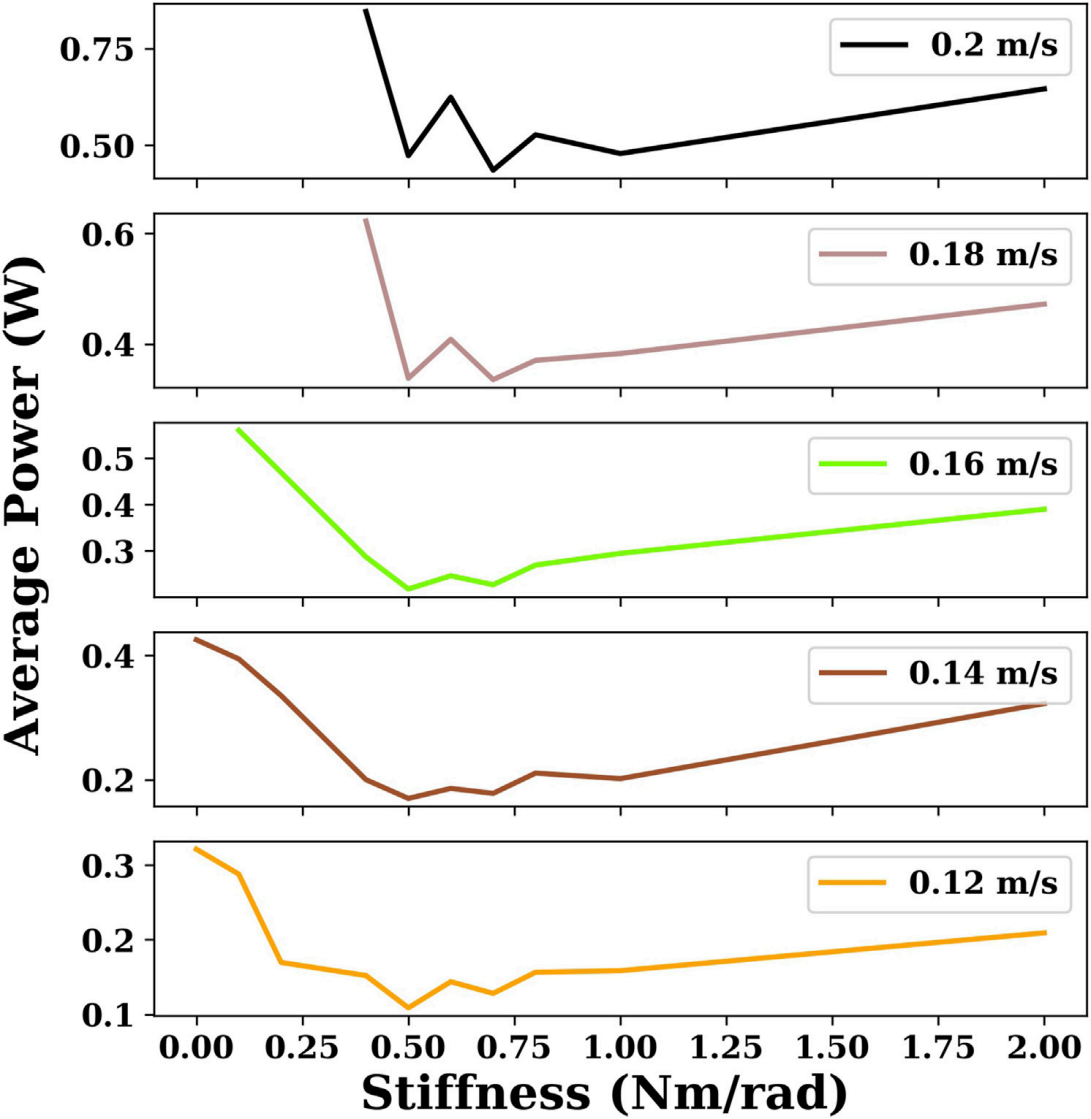
This plot indicates the minimum power consumption for the given velocities for snake robots with different joint stiffnesses using the gait equation controller. There are some cases of joint stiffness for which there are no sample points when the velocity is large because the maximum velocity of the snake robot is smaller than the given velocity.

In Figure 4, six given velocities are chosen to compare the energy efficiency of snake robots with different joint stiffnesses using the gait equation controller. As shown in Figure 4, when the velocity is small (0.04 m/s), the increase in joint stiffness results in an increase in energy consumption. However, when the velocity becomes larger, the snake robot with an appropriate joint stiffness (e.g., 0.5 Nm/rad) is more energy-efficient than the snake robot with no joint stiffness (joint stiffness is 0).

**FIGURE 5:**
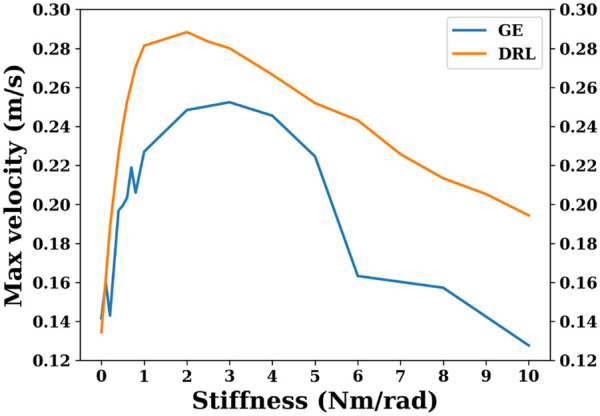
This plot shows maximum velocities that can be achieved by snake robots with different joint stiffnesses using the gait equation controller or DRL controller.

Figure 5 shows the maximum velocity that can be achieved by a snake robot with different joint stiffnesses using the gait equation controller or DRL controller. As shown in Figure 5, within a certain range, the maximum velocity that can be achieved by the snake robot is increased as the joint stiffness increases. However, after a certain range, increasing the joint stiffness will increase the energy consumption and decrease the maximum velocity of the snake robot. The snake robot using the gait equation controller has the best energy efficiency when the joint stiffness is 0.5 Nm/rad. The maximum velocity that can be achieved by the snake robot using the gait equation controller is largest when the joint stiffness is 3 Nm/rad.

### 4.2 Result of the DRL controller

**FIGURE 6:**
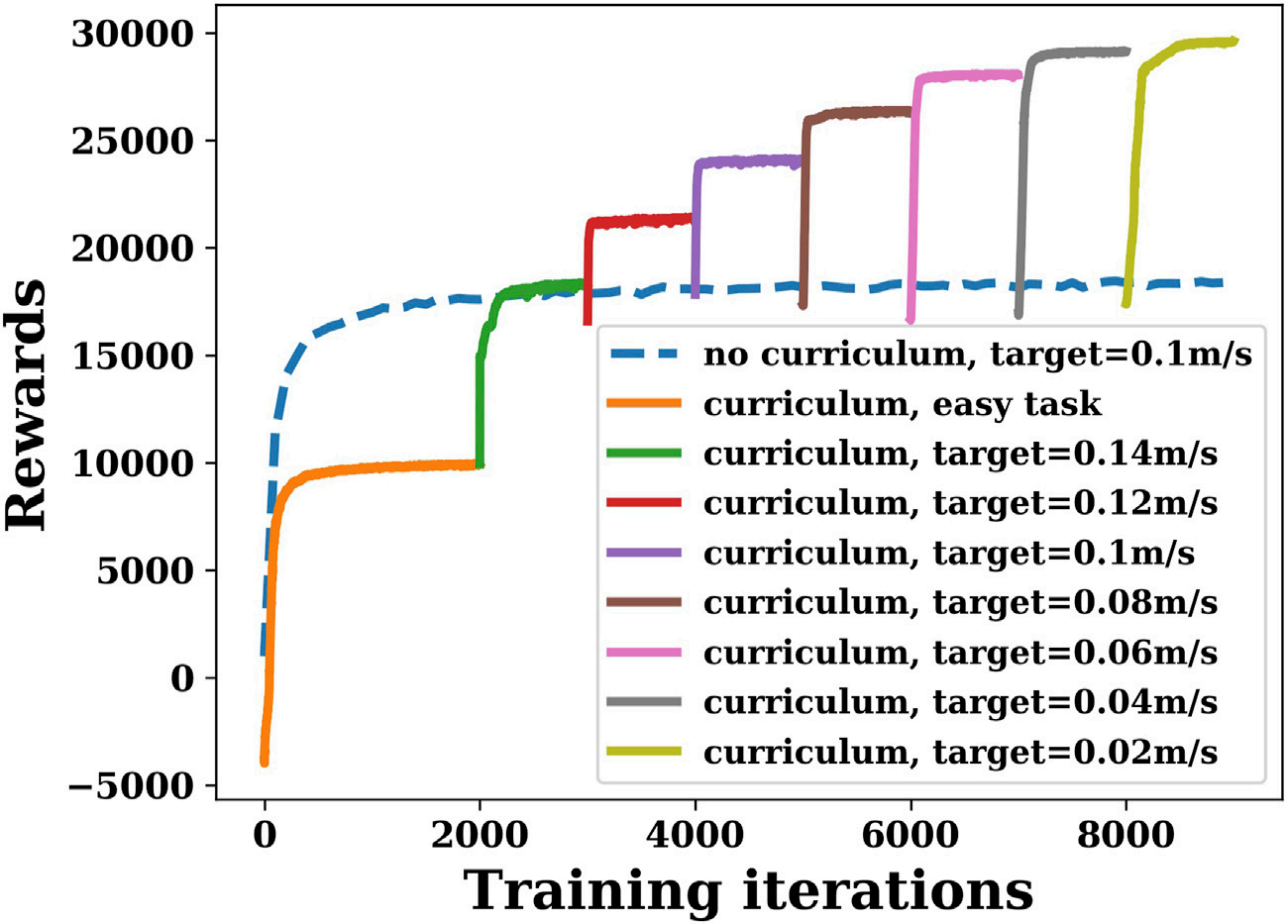
Deep reinforcement learning training rewards with or without curriculum learning when joint stiffness is 0.

The reward curve of the DRL controller is shown in Figure 6. As we mentioned in the previous section, in the first 2,000 episodes, the reward is set as function *r*_2_. After 2,000 episodes, the reward changes to function *r*_1_, and the target velocity decreases by 0.02 m/s every 1,000 episodes, starting from 0.14 m/s and decreasing to 0.02 m/s. To demonstrate that curriculum learning is necessary, Figure 6 also shows the result without curriculum learning. The blue dashed line represents the training results using the reward function *r*_1_ directly in the first 2,000 episodes, in which the target velocity was set to 0.1 m/s. The other colored lines show the results of curriculum learning. It can be seen that the final reward curve converges as the number of training iterations increases, regardless of the reward function used. However, by comparing the two cases (blue and purple) with the same target velocity of 0.1 m/s (where the reward functions are identical in both cases), it can be seen that if the robot is trained directly with a more complex function *r*_1_, although the reward curve converges, the final reward is much lower than that using curriculum learning.

**FIGURE 7:**
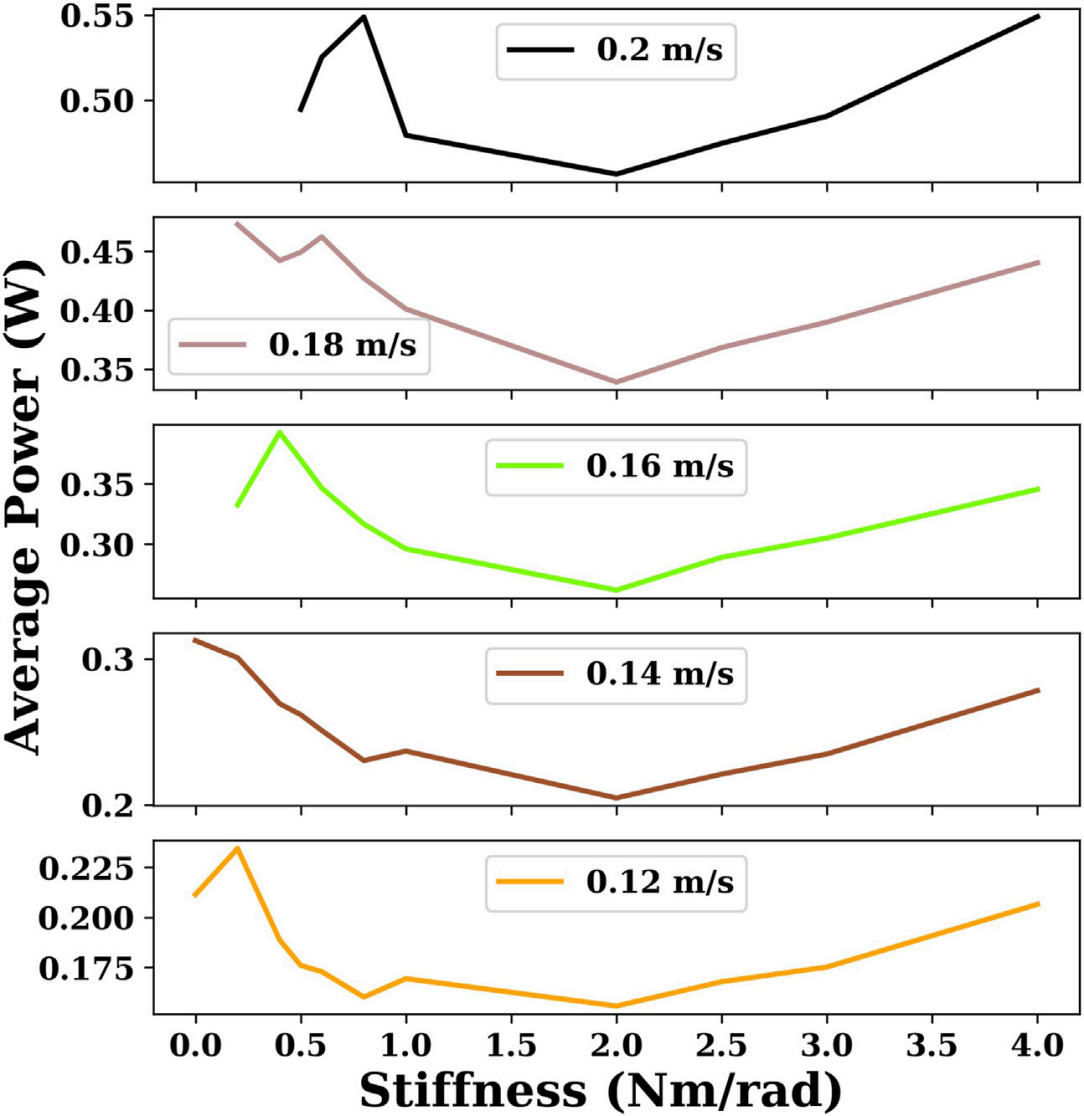
This plot indicates the minimum power consumption for the given velocities for snake robots with different joint stiffnesses using the DRL controller. There are some cases of joint stiffness for which there are no sample points when the velocity is large because the maximum velocity of the snake robot is smaller than the given velocity.

For each stiffness condition, we obtained results for multiple target velocities ranging from 0.02 m/s to the maximum velocity with a step of 0.02 m/s. In Figure 7, six velocities are chosen to compare the energy efficiency of snake robots with different joint stiffnesses with DRL.

As can be seen in Figure 5 and Figure 7, the overall global trend is similar to that of the gait equation controller; as the joint stiffness increases over a range, the energy efficiency and the maximum velocity of the snake robot are improved. However, beyond a threshold, the increase of joint stiffness has an opposite effect, and the snake robot becomes more and more energy-intensive. For the DRL controller, when the joint stiffness is 2 Nm/rad, the snake robot is the most energy-efficient and can reach the largest maximum velocity of nearly 0.3 m/s.

**FIGURE 8:**
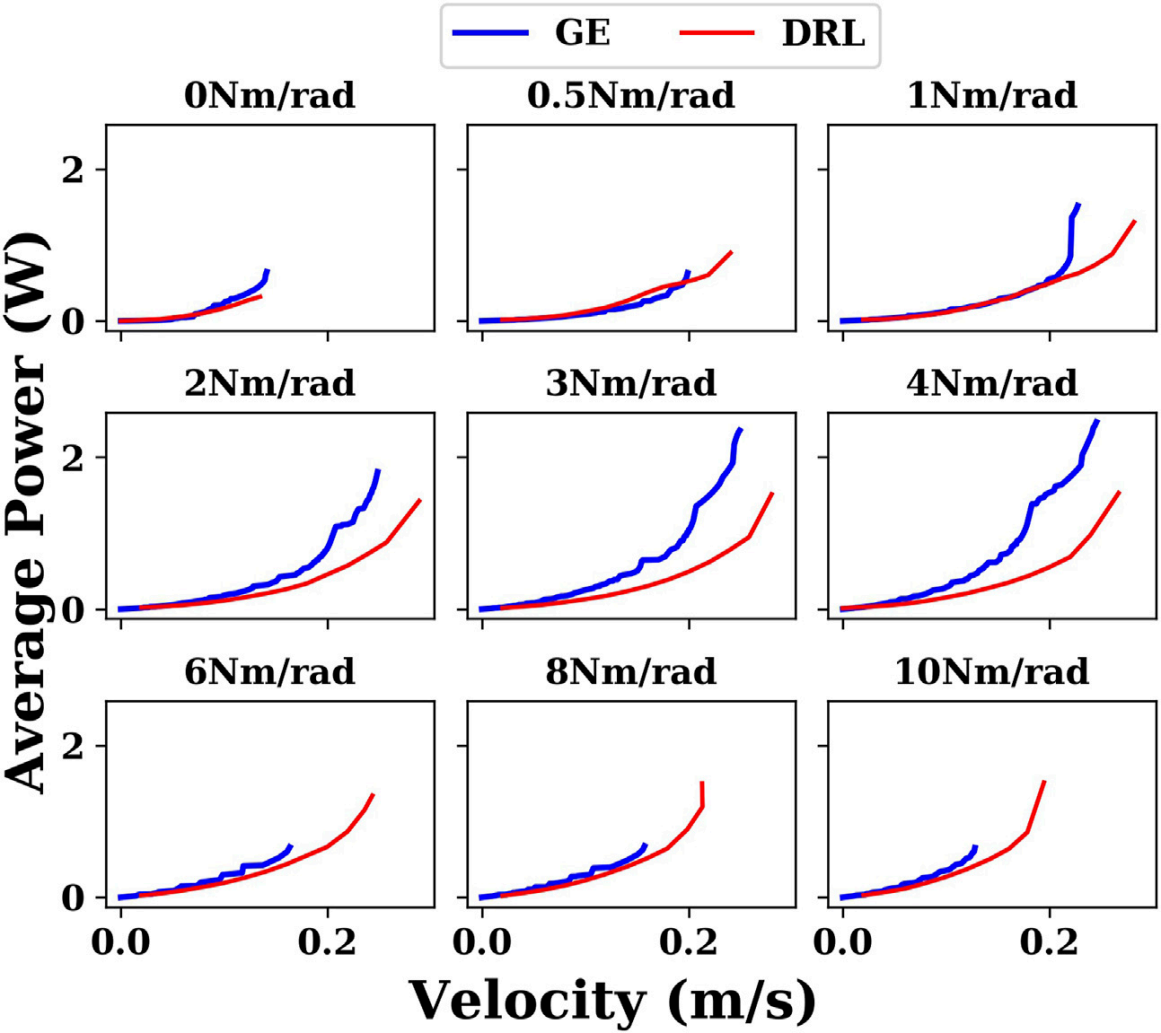
Comparison of the results generated by the gait equation controller and DRL controller with different joint stiffnesses in the water environment.

Figure 8 plots the energy results generated by the gait equation and DRL controller with different joint stiffnesses in a water environment. It can be seen that in almost all cases, the results of the DRL controller are better than those of the gait equation controller.

### 4.3 Adaptation to different liquid environments

**FIGURE 9:**
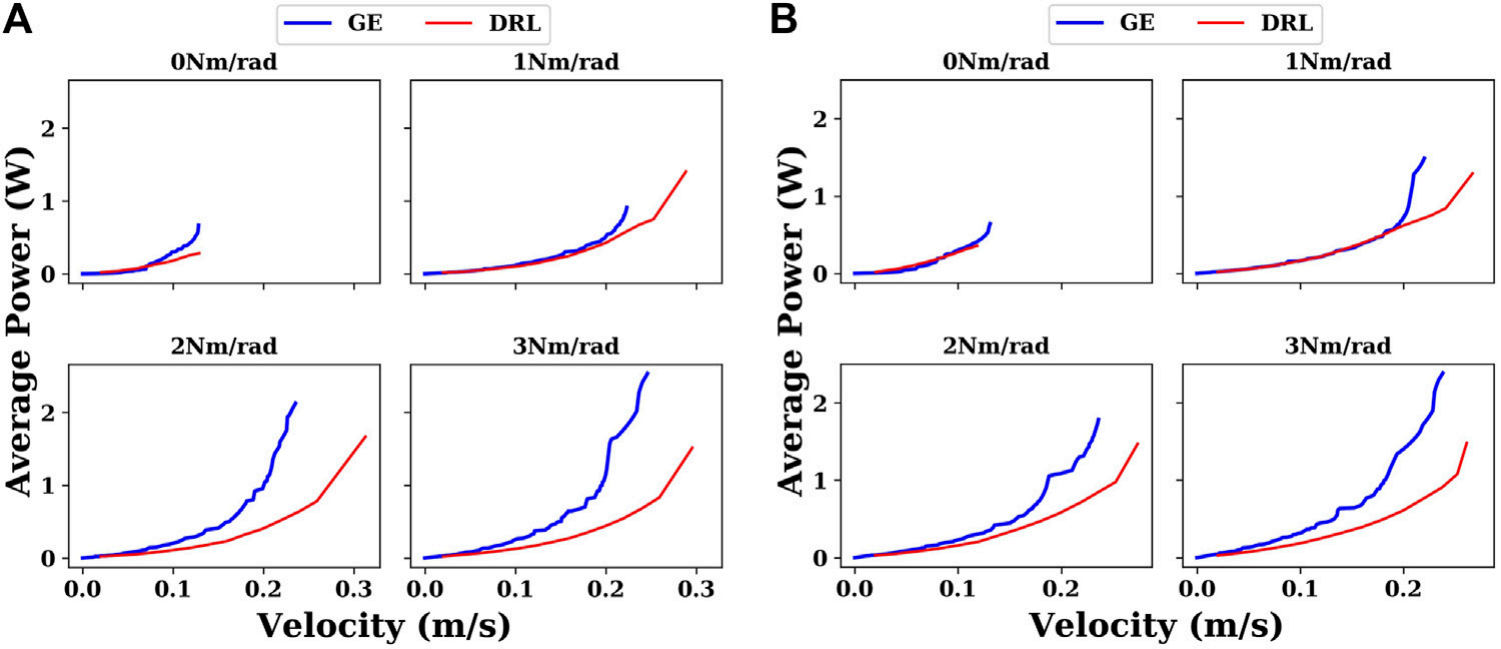
Comparison between the gait equation controller and DRL controller with different joint stiffnesses in different liquid environments. **(A)** In propylene (density of 514 kg/m^3^ and viscosity of 0.0001 Pa⋅ s); **(B)** in ethylene glycol (density of 1,097 kg/m^3^ and viscosity of 0.016 Pa⋅ s).

Sometimes, snake robots do not just work in water, but in extreme situations, such as when there is a marine oil spill, where snake robots need to work in different liquid environments, it is necessary that the control methods can be adapted to these extreme situations. So we have also carried out experiments in different liquid environments. We are primarily concerned with viscosity. Because the densities of common liquids vary but are of the same order of magnitude, they do not have a significant effect on the motion of the snake robot. Viscosity, however, can vary by orders of magnitude, for example, in the case of gas-free crude oil, which can have a viscosity over 1 Pa⋅ s Beal (1946). As shown in Figure 9, in different liquid environments, similar results were obtained as in water. The DRL controller demonstrates its adaptability and superiority compared with the gait equation controller in various environments.

## 5 Discussion

### 5.1 Comparison of the gait equation and DRL controller

As we mentioned in the previous section, either the gait equation controller or the DRL controller can improve the robot’s energy efficiency within a certain threshold range as long as the joint stiffness is increased. However, we notice some differences between the gait equation and DRL results. DRL succeeds to find more performant control solutions in terms of energy efficiency and also maximum speed. The optimal stiffness setting found was 2 Nm/rad in contrast to 0.5 Nm/rad of the gait equation. This optimal stiffness difference would come from the difference of the spatio-temporal pattern of the control input. The GE controller employs the traveling sine wave signals; there are still assumptions for the waves which can be considered. For example, the neighboring joint oscillation frequency is assumed to be same. The GE controller approach is used for its simplicity, but its solution space is on the traveling sine waves. In turn, DRL control can potentially apply out of this solution space. Then, it indicates that DRL must succeed in finding a better way to actively use the joint elasticity and taking advantage of spring-stored energy for optimizing the total swimming energy. This result can be interpreted from a mechanic’s point of view as follows. A certain degree of joint stiffness can realize a type of swimming that stores potential energy so that the stiffness works positively in terms of total energy to the extent that the stored energy can be well utilized. However, if the body is too stiff, the total energy can be too high because the energy is wasted for bending the body joints itself. Therefore, it was quantitatively demonstrated that there is a trade-off relationship between the energy efficiency of swimming and the body stiffness.

**FIGURE 10:**
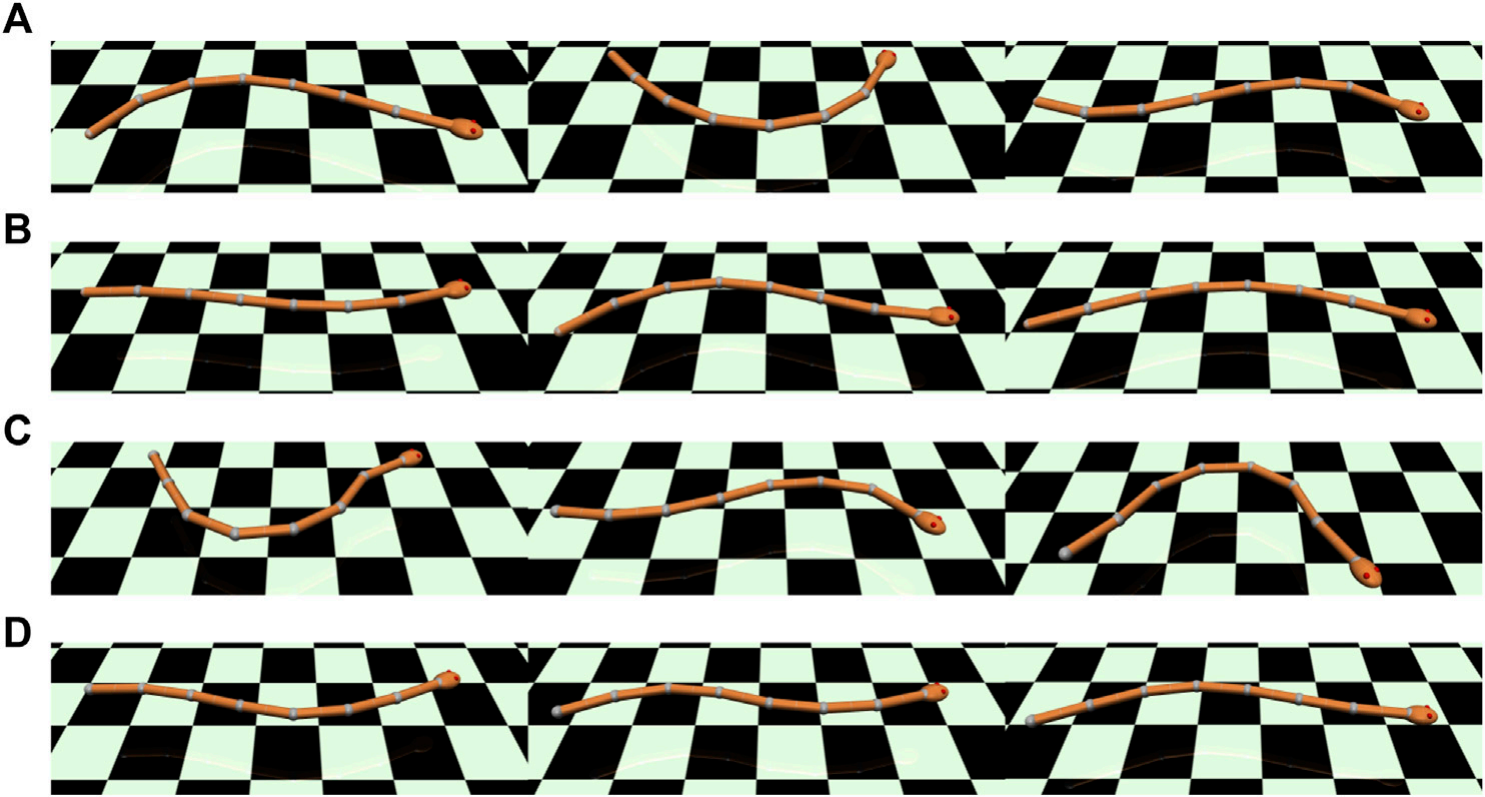
Montages show the swimming posture of the snake-like robot under different conditions. The frames are sorted in four columns from top to bottom and are recorded at intervals of 0.4s. Please refer to the video associated with the article. **(A)** Gait GE0: joint stiffness = 0 Nm/rad with the gait equation controller; velocity = 0.117 m/s; average power = 0.332 W. **(B)** Gait GE2: joint stiffness = 2 Nm/rad with the gait equation controller; velocity = 0.201 m/s; average power = 0.856 W. **(C)** Gait DRL0: joint stiffness = 0 Nm/rad with the DRL controller; velocity = 0.124 m/s; average power = 0.309 W. **(D)** Gait DRL2: joint stiffness = 2 Nm/rad with the DRL controller; velocity = 0.199 m/s; average power = 0.455 W.

**FIGURE 11:**
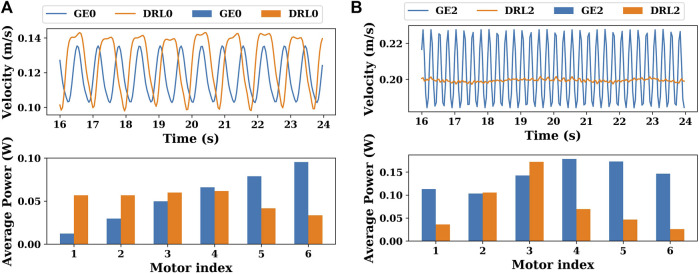
**(A)** Variation of the CoM velocities and the energy consumption of each joint of gaits GE0 and DRL0 (joint stiffness = 0 Nm/rad). **(B)** Variation of the CoM velocities and the energy consumption of each joint of gaits GE2 and DRL2 (joint stiffness = 2 Nm/rad).

Figure 10 shows the gaits of the snake robot with the gait equation controller or DRL controller. As shown in Figure 10, the four gaits will be abbreviated in the following as GE0, DRL0, GE2, and DRL2. “GE” and “DRL” mean that the gait is generated by the gait equation controller or the DRL controller. “0” and “2” mean that the robot has a joint stiffness of 0 or 2 Nm/rad. As shown in Figure 11, in order to better compare the differences between these gaits, we plot the variation of the CoM velocity of the snake robot and the energy consumption of each joint for these four gaits.

When the joint stiffness is 0 Nm/rad, as shown in Figure 11A, the amplitude of the variation of the CoM velocity of the DRL0 gait is slightly greater than that of the GE0 gait, but the frequency is less than that of the gait generated by the gait equation controller (between 16 and 24 s, the DRL0 gait has 8 peaks in velocity, while the GE0 gait has 12 peaks in velocity). In terms of the energy consumption of the actuator, the GE0 gait is more like an eel-like motion gait, i.e., the energy consumption becomes progressively greater from the head to tail. The energy consumption of the DRL0 gait is mainly distributed in the front half of the snake. In this case, the energy efficiency of the DRL0 gait is marginally better than that of the GE0 gait.

When the joint stiffness is 2 Nm/rad, it can be seen in Figure 11B that the velocity variation of the GE2 gait is still large, which is also due to the characteristics of the gait equation controller itself. In contrast, the velocity of the DRL2 gait has almost no fluctuations, and the snake robot can move at a very steady velocity. The energy consumption of the actuators is higher for each actuator in the GE2 gait, and the energy consumption of the second half of the snake (motor index = 4, 5, 6) is slightly higher than the first half (motor index = 1, 2, 3). In contrast, the energy consumption of the DRL2 gait was mainly concentrated in the third joint. It indicates that DRL employed different swimming modes when it can take advantage of joint elasticity as motor adaptation to the given body condition.

## 6 Conclusion

We propose a gait design method for an underwater snake robot based on DRL and curriculum learning, especially for taking advantage of joint elasticity toward energy efficiency. We demonstrate that the DRL controller can produce a more energy-efficient gait than the gait equation controller in most cases. The results demonstrate that employing appropriate elasticity to the articulated joints can effectively reduce the energy consumption during locomotion for both the gait equation controller and DRL controller. The energy efficiency itself comes from the body stiffness; however it is another issue if we can find the spatio-temporal control pattern which can take advantage of it, even if they have the same body property. The comparison demonstrates that the DRL controller can manage to find the pattern of control which maximizes the effect. Moreover, an appropriate joint elasticity can increase the maximum velocity achievable by the snake robot underwater. Experiments in different liquid environments confirm that the DRL controller’s adaptability is superior to the gait equation controller.

We believe that joints with some degree of stiffness can resemble the characteristics of snakes in nature, possibly increasing the robots’ dynamic performance. This study can contribute to the design of the energy-efficient gait for underwater snake robots and the understanding of the joint elasticity effect. In addition, the energy-efficient gait can help snake robots to operate for longer periods in underwater environments with limited energy resources. This article focused on the aspect of the joint elasticity of rigid body connections to improve the energy efficiency. It can be interesting to study some other aspects as well for future studies as a natural living system is well designed to have energy efficiency with many other factors such as body softness, body form, and control system-like spiking neural networks Naya et al. (2021).

In this study, we only tested forward locomotion. In future works, we will consider different types of gait behaviors, such as turning, accelerating, and three-dimensional movements. In addition, we will explore the influence of joint elasticity on other types of movements.

## Data Availability

The raw data supporting the conclusions of this article will be made available by the authors, without undue reservation.
